# A survey on the willingness of outpatients to participate in fundus examination procedures conducted by ophthalmology training residents in China: A cross‐sectional study

**DOI:** 10.1002/hsr2.1870

**Published:** 2024-02-13

**Authors:** Haidong Li, Tiankun Li, Yuanyuan Fan, Bin Zheng, Yun‐e Zhao

**Affiliations:** ^1^ National Clinical Research Center for Ocular Diseases, Eye Hospital Wenzhou Medical University Wenzhou China; ^2^ Tianjin Key Laboratory of Ophthalmology and Visual Science, Tianjin Eye Institute, Tianjin Eye Hospital, School of Medicine Nankai University Tianjin China

**Keywords:** doctor–patient relationship, fundus examination, ophthalmology training, standardized residency training

## Abstract

**Background and Aims:**

The National Standardized Training for Resident Doctors (STRD) in mainland China encounters many challenges in its implementation. To investigate whether outpatients are willing to undergo indirect ophthalmoscopy examination conducted by ophthalmology residents in the ophthalmology STRD program in China.

**Methods:**

This study conducted a cross‐sectional survey at the Eye Hospital of Wenzhou Medical University between September 2021 and September 2023. A cohort of 300 initial outpatients requiring indirect ophthalmoscopy examinations were enlisted from the outpatient department. Based on whether the patients are willing to undergo an indirect ophthalmoscopy examination by resident doctors, patients were divided into two groups: Group 1 (willing) and Group 2 (unwilling), and their questionnaire responses were comparatively analyzed.

**Results:**

A total of 261/300 (87%) valid questionnaires were returned in the survey, which included 149 males and 112 females. No notable gender difference (*p* = 0.400) or disparity in medical expense categories (*p* = 0.786) was observed between the two groups. However, variables such as outpatient marital status (*p* = 0.002), the presence of training faculty during fundus examinations with residents and outpatients (*p* < 0.001), the demeanor of training residents toward patients (*p* < 0.001), and the quality of doctor–patient communication (*p* < 0.001) significantly varied between the groups.

**Conclusion:**

The level of outpatients' cooperation with ophthalmology residents during fundus examinations in the Chinese ophthalmology STRD program was observed to be low. Enhancing the presence of training faculty during examinations and enhancing the communication skills of training residents could significantly improve this situation.

## 1 BACKGROUND

Training proficient and capable ophthalmic professionals is a challenging endeavor, especially in a vast country like China.^1^ Globally, ophthalmology residency programs vary in their approach, with several countries lacking quality control mechanisms for ophthalmologists or their respective programs.^2^ In mainland China, the implementation of the National Standardized Training for Resident Doctors (STRD) in 2014 aimed to enhance training quality and elevate ophthalmologists' capabilities to align with international standards.^3^ This program acts as a crucial link between medical education and a career in ophthalmology, significantly influencing the enhancement of ophthalmic healthcare quality. Notably, fundus diseases are a primary cause of visual impairment, necessitating an elevation in clinical diagnostic and treatment standards in China. The unique anatomy of the fundus requires specialized instruments for examination, posing complexities in clinical teaching and presenting significant challenges for ophthalmology residents.^4^ Furthermore, regional disparities in training programs due to the lack of a unified national standard have resulted in varying capabilities among graduating ophthalmology residents in China.^1^ The continuous evolution of the Chinese ophthalmology STRD program has seen significant improvements over the years, including advancements in curriculum design, incorporation of modern technology in training modules, and an enhanced emphasis on practical clinical experiences. These changes underscore the program's adaptability and responsiveness to contemporary challenges in ophthalmology training.

In the past two decades, patient distrust in doctors has surged in mainland China, leading to strained doctor–patient relationships and diminished overall trust.^5^, ^6^ Certainly, the tense doctor–patient relationship in mainland China stems from various factors contributing to broader societal issues. The communication breakdowns between doctors and patients are due to a lack of effective communication strategies or inadequate patient education about medical conditions and treatments. Challenges in healthcare accessibility, especially in rural areas, lead to disparities in medical resources and services. This could result in dissatisfaction and frustration among patients seeking healthcare. Understanding these multifaceted issues helps provide context to the strained doctor–patient relationship in China and underscores the significance of effective communication, improved healthcare accessibility, cultural competence, and enhanced trust‐building strategies within the healthcare system.

Outpatients in the department exhibit high mobility and brief stays, making it challenging to establish a consistent doctor–patient relationship. However, fostering a trusting rapport is pivotal for top‐notch medical care. Yet, consequently, ophthalmology residents participating in the STRD program often face patient rejections while treating fundus disease cases in outpatient settings. This not only dampens residents' enthusiasm but also hampers the effectiveness and quality of ophthalmology training. As both doctors and patients frequently move, better standardization in ophthalmic education within outpatient settings is imperative. To elevate the quality of ophthalmology residency training, enhancements in teaching effectiveness and assessment methods are necessary. This study aims to explore patients' willingness to collaborate with ophthalmology residents during indirect ophthalmoscopy examinations as part of the ophthalmology STRD program in China. It further seeks to analyze influencing factors and propose corresponding solutions.

## 2 METHODS

This study was a cross‐sectional survey conducted using a self‐administered questionnaire, which received approval from the Eye Hospital of Wenzhou Medical University in Zhejiang Province, China. The survey spanned from September 2021 to September 2023 and employed convenience sampling to recruit outpatients from the outpatient department at the Ophthalmology Standardized Residency Training Base in the Eye Hospital of Wenzhou Medical University.

Participants eligible for the study were outpatients requiring their first visit for an indirect ophthalmoscopy examination, within the age range of 18–60 years. The selected age range was based on the need for ensuring participants who were legally capable of providing consent and understanding the nature of the examination. In addition, limiting the age range helps ensure a more homogeneous sample, enhancing the internal validity of our findings and allowing for more focused analyses within the target population. Follow‐up outpatients and inpatients were excluded from participation. Researchers provided standardized explanations about the survey's purpose using a scripted format. In the survey, outpatient participants provided verbal consent and completed the questionnaire in the waiting room after being informed that participation did not involve compensation and that refusal to participate would not incur any penalties. The questionnaire responses of the outpatient participants were kept confidential from the ophthalmology residents and faculty.

First, outpatients watch an instructional video explaining the procedure of indirect ophthalmoscopy examination, gaining a comprehensive understanding of the examination's procedure, necessity, and potential discomfort. Subsequently, they were asked if they were willing to undergo the examination conducted by the ophthalmology training residents, then were divided into Group 1 (Willing) and Group 2 (Unwilling) based on their willingness. The questionnaire was designed using methods outlined in previously published literature.^7^, ^8^ This questionnaire was designed to obtain information on the following five categories: (1) demographic information (gender and age), (2) medical expense categories, (3) presence of training faculty during fundus examinations by residents for outpatients, (4) attitudes of training residents toward patients, and (5) adequacy of doctor–patient communication. The response format was binary, allowing participants to choose either “Yes” or “No.” This choice of format aimed to simplify the response process for participants to ensure clarity and ease of understanding. All responses were verified and invalid questionnaires were excluded. Invalidation criteria encompassed: (1) unclear or incomplete responses, (2) multiple answers to single‐choice questions, and (3) duplicate questionnaires. The questionnaire was designed for self‐completion and typically took around 10 min to finish. Participants were directed to independently complete the questionnaire.

Statistical analysis was performed using SPSS 23.0 statistical software (IBM SPSS, version 23; IBM Corp.). The continuous data for age are presented as mean ± standard deviation and were compared using Student's *t* test. Categorical variables for all other factors were presented as frequencies and proportions and analyzed using the *χ* ^2^ test. The *p* < 0.05 was considered statistically significant and the tests were two‐sided.

## 3 RESULTS

In the survey, a total of 261/300 (87%) valid questionnaires were returned, including 149 (57.1%) males and 112 (42.9%) females. The average age of all participants was 40.7 ± 11.7 years (range: 18–60). These individuals were categorized into two groups: Group 1 comprised 125 patients (47.9%, 125/261), while Group 2 included 136 patients (52.1%, 136/261). A comparative analysis of various factors between the groups is presented in Table 1. Gender (*p* = 0.400) and the category of medical expenses (*p* = 0.786) exhibited no significant differences between the two groups. However, variables like marital status (*p* = 0.002), the presence of training faculty during fundus examinations with residents and outpatients (*p* < 0.001), training residents' attitudes towards patients (*p* < 0.001), and the quality of doctor–patient communication (*p* < 0.001) significantly varied. Factors such as unmarried marital status among outpatients, the attendance of training faculty during fundus examinations, positive attitudes from training residents towards patients, and effective doctor–patient communication positively influenced outpatients' willingness to collaborate with ophthalmology training residents during fundus examinations.

**Table 1:** A comparison of outpatients' willingness to cooperate with ophthalmology training residents during fundus examination in Chinese STRD program.

Item	Group 1	Group 2	*t*/*χ* ^2^	*p* Value
	*n* = 125 (%)	*n* = 136 (%)		
Age (years)	39.3 ± 11.9	42.1 ± 11.5	−1.900	0.059
Gender				
Male	68 (54)	81 (60)	0.708	0.400
Female	57 (46)	55 (40)		
Marital status				
Married	74 (59)	55 (40)	9.169	0.002
Unmarried	51 (41)	81 (60)		
Types of medical expenses				
Medical insurance	65 (52)	73 (54)	0.073	0.786
Self‐funded	60 (48)	63 (46)		
Faculty being present at the fundus examination site together with training residents				
Yes	101 (81)	39 (29)	71.161	<0.001
No	24 (19)	97 (71)		
Training residents' attitudes towards outpatients				
Good	99 (79)	35 (26)	74.527	<0.001
Bad	26 (21)	101 (74)		
Doctor–patient communication being sufficient				
Yes	88 (70)	44 (32)	37.720	<0.001
No	37 (30)	92 (68)		

*Note*: Continuous data for age are expressed as mean ± standard deviation and were compared using the Student's *t* test. Categorical variables for all other variables were expressed as frequencies and proportions and were compared using the *χ* ^2^ test.

Abbreviation: STRD, Standardized Training for Resident Doctors.

## 4 DISCUSSION

The survey found that several factors influenced outpatients' willingness to cooperate with ophthalmology training residents during fundus examinations in the Chinese STRD program. These factors included the patients' marital status, the presence of training faculty during examinations, the attitudes of training residents toward patients, and the quality of doctor–patient communication. Patients showed a significantly higher willingness to cooperate when training faculty were present alongside residents, compared to their absence. Consequently, the involvement of training faculty in clinical ophthalmology teaching plays a crucial role in the successful execution of the standardized residency training program. Internationally, the quality of ophthalmology residency training programs varies widely, with most countries lacking accreditation guidelines for supervision.^2^ In mainland China, the standardized training system for ophthalmology residents was established relatively late, resulting in a notable gap compared to developed nations. Increased commercialization and reduced consultation time between patients and doctors have limited the time available for training faculty to teach in hospital outpatient clinics.^9^ To ensure high‐quality completion of standardized residency training in ophthalmology for fundus diseases, a comprehensive assessment system is imperative. This system should encompass evaluations not only for ophthalmology residents but also for the training faculty. Establishing detailed evaluation plans for teaching effectiveness and faculty quality within the ophthalmology STRD program is crucial. This may involve daily assessments, efficient time management, one‐on‐one mentoring of residents, and a fixed daily teaching schedule, among other measures.

Numerous challenges hinder ophthalmology residents from conducting thorough fundus examinations on patients. The intricate nature of fundus diseases arises from the fundus tissue's internal position within the eye, necessitating specialized instruments and patient cooperation for diagnosis and treatment. Patients might experience discomfort due to prolonged exposure to bright light during procedures like indirect ophthalmoscopy, discouraging their participation in examinations led by residents. This research underscores that patients are more inclined to undergo fundus examinations conducted by ophthalmology residents when these residents communicate effectively and exhibit positive attitudes. The Accreditation Council for Graduate Medical Education (ACGME) outlines six core competencies essential for physicians, emphasizing not just medical knowledge but also interpersonal communication skills.^2^ In ophthalmology residency training, the importance of effective communication skills is increasingly recognized as a pivotal competency. Patients' trust in their doctor's compassion significantly influences their assessment of the doctor–patient relationship, surpassing their trust in the doctor's expertise.^5^, ^10^ Implementing robust doctor–patient communication, coupled with positive attitudes, fosters a more amicable relationship between ophthalmology residents and outpatients with fundus diseases. This, in turn, creates valuable learning opportunities for residents within the standardized residency training in ophthalmology.

In comparison to the detailed curriculum requirements in US ophthalmology residency training, where formal lectures are a core component, Chinese curriculum requirements are comparatively less specific.^1^ The extensive clinical workload in Chinese standardized residency training has hindered the implementation of formal teaching sessions. Studies have indicated lower satisfaction among Chinese ophthalmology residents, partly due to excessive time spent on administrative tasks and insufficient supervision.^11^ The “softer” interpersonal aspects such as caring, appreciation, and empathy have been found to be important to patients in patient–doctor relationships.^12^ Research underscores the pivotal role of patient trust in a doctor's compassion and empathy in establishing trusting doctor–patient relationships. These elements significantly contribute to the development of harmonious and trustful interactions between healthcare providers and patients.^5^ In standardized residency training, both interpersonal and clinical skills hold equal importance. Emphasizing doctors' empathy is particularly vital in cultivating trust and building positive doctor–patient relationships. In addition, the results of this study indicate that unmarried outpatient patients show lower willingness to cooperate, and most of them are young individuals. This is an intriguing observation. With the rapid advancement of information technology, the use of smartphones in China has become widespread, and self‐media information is prevalent. Some information might intentionally exaggerate or even fabricate medical conflicts to attract more online traffic. Younger individuals are more susceptible to the influence of public opinion. This suggests that China's healthcare regulatory authorities should strengthen positive public opinion guidance and leverage online media to facilitate the resolution of doctor–patient conflicts.

Our survey faces several limitations. Primarily, the small sample size and the exclusion of patients under 18 years and over 60 years might compromise the survey's representativeness. In addition, selective bias was present as the survey excluded outpatient follow‐up patients and inpatients. The questionnaire's design lacked information on patients' education and income levels, imposing constraints on the survey's content. Finally, the questionnaire was not validated and the cross‐sectional design limited our ability to establish definitive causal relationships. The potential biases introduced by convenience sampling could limit the study's ability to represent diverse perspectives, potentially affecting the external validity of the findings. The findings presented in this paper represent our preliminary work, and our next steps involve delving deeper into these areas of concern.

In conclusion, our study unveiled a low willingness among outpatients to cooperate with training residents during fundus examinations in the Chinese ophthalmology STRD program. The results underscore the significance of training faculty presence, positive attitudes of training residents, and effective doctor–patient communication in enhancing outpatients' cooperation with training residents in the Chinese ophthalmology STRD program.

## AUTHOR CONTRIBUTIONS

All authors have read and approved the final version of the manuscript.

## CONFLICT OF INTEREST STATEMENT

The authors declare no conflict of interest.

## TRANSPARENCY STATEMENT

The lead author Bin Zheng, Yun‐e Zhao affirms that this manuscript is an honest, accurate, and transparent account of the study being reported; that no important aspects of the study have been omitted; and that any discrepancies from the study as planned (and, if relevant, registered) have been explained.

## Data Availability

The data that support the findings of this study are available from the corresponding authors upon reasonable request. The authors confirm that the data supporting the findings of this study are available within the article.
